# Coexistence of 21-hydroxylase deficiency and Gitelman syndrome in a neonate presenting with severe hyponatremic seizures: a case report

**DOI:** 10.3389/fendo.2026.1841630

**Published:** 2026-06-10

**Authors:** Ruiping Zhang, Ying Zhang, Yanyan Nie, Yanling Bai, Jianbo Shu, Yang Liu

**Affiliations:** 1 Neonatal Department of Longyan Division, Tianjin University Children’s Hospital/Tianjin Children’s Hospital, Tianjin, China; 2 Tianjin Pediatric Research Institute, Tianjin Key Laboratory of Birth Defects for Prevention and Treatment, Tianjin University Children’s Hospital/Tianjin Children’s Hospital, Tianjin, China; 3 The Pediatric Clinical College of Tianjin Medical University, Tianjin, China

**Keywords:** 21-hydroxylase deficiency, convulsion, Gitelman syndrome, neonate, severe hyponatremia

## Abstract

**Introduction:**

21-hydroxylase deficiency is an autosomal recessive genetic disease caused by congenital deficiency of 21-hydroxylase. Gitelman syndrome (GS) is a rare autosomal recessive renal tubular disease. The coexistence of these two diseases in the same patient is rare in clinical practice.

**Methods:**

We report the case of a newborn with concurrent 21-hydroxylase deficiency and GS who presented with severe hyponatremic seizures. We describe the clinical manifestations, physical examination findings, laboratory results, treatment, and follow-up.

**Results:**

A male newborn was admitted to the neonatal intensive care unit at Tianjin Children’s Hospital for intermittent convulsions for 10 days. Laboratory tests showed severe hyponatremia, with a serum sodium of 88 mmol/L, which was considered the cause of the convulsions. Further testing showed markedly elevated 17-hydroxyprogesterone (17-OHP) and adrenocorticotrophic hormone (ACTH) levels (17-OHP, 740.2 ng/mL; reference range, <3 ng/mL; ACTH, 488.4 pg/mL; reference range, 7.2–63.3 pg/mL). Therefore, 21-hydroxylase deficiency was clinically diagnosed. Serum potassium was mostly within the normal range, but hypomagnesemia was recorded. Whole-exome sequencing and multiplex ligation-dependent probe amplification combined with long-range PCR were performed. There were two pathogenic variants identified, a homozygous variant of 293-13C>G (p.)? in intron 2 in the *CYP21A2* gene and a compound heterozygous variant of c.473G>A (p.Arg158Gln) and c.2720 + 3A>T (p.)? in the *SLC12A3* gene. Genetic testing confirmed two rare diseases in this infant: 21-hydroxylase deficiency and Gitelman syndrome.

**Conclusion:**

The coexistence of 21-hydroxylase deficiency and GS in a neonate has rarely been reported. These two pathologies determine the opposite values of potassium (hyperkalemia in 21-hydroxylase deficiency and hypokalemia in GS). This case report provides diagnostic and treatment insights for clinicians and highlights the value of genetic testing when the diagnosis is uncertain.

## 1 Introduction

Neonatal convulsions are a common clinical symptom, commonly caused by intracranial infection, intracranial hemorrhage, neonatal hypoxic-ischemic encephalopathy, electrolyte disturbances, hypoglycemia, epilepsy, and inherited metabolic diseases (1, 2). Electrolyte disturbances mainly include hyponatremia, hypocalcemia, and hypomagnesemia. Hyponatremia, defined as a serum sodium concentration less than 135 mmol/L, is the most common electrolyte disorder in hospitalized children and can cause seizures in infants (2, 3). We report a case of neonatal seizures caused by severe hyponatremia.

Congenital adrenal hyperplasia (CAH) is a group of autosomal recessive disorders caused by congenital defects in the enzymes required for the synthesis of corticosteroids. The incidence of CAH ranges from 1:10,000 to 1:20,000 (4, 5). 21-hydroxylase deficiency (21-OHD) is the most common type, accounting for 90% to 95% cases (4). It is characterized by adrenal insufficiency, hyperandrogenism, virilization, and growth failure.

Gitelman syndrome (GS) is a rare autosomal recessive renal tubular disease, characterized by hypokalemia, metabolic alkalosis, hypomagnesemia, and hypocalciuria. The prevalence of GS is approximately 1–10 per 40,000 people and may be higher in Asian populations (6). GS typically presents in adolescents and adults but is rarely reported in neonates (6, 7). 21-OHD has typical clinical manifestations in the neonatal period, while GS has no obvious clinical manifestations in the neonatal period.

This article describes a case of severe hyponatremia presenting with seizures as the main manifestation in the neonatal period. The underlying cause of electrolyte disturbance was two rare diseases, 21-OHD and GS. To our knowledge, no neonatal cases of concurrent 21-OHD and GS have been previously reported. This article aims to provide clinical experience for clinicians by describing the clinical manifestations, findings from physical and laboratory examinations, treatment, and follow-up.

## 2 Case presentation

A male newborn was admitted to the neonatal intensive care unit at Tianjin Children’s Hospital due to intermittent convulsions for 10 days. There was no history of vomiting or diarrhea. On physical examination, the temperature was 36.5°C, blood pressure was 65/40 mmHg, pulse rate was 130 beats per minute, and body weight was 3,340 g. The examination also showed growth retardation (birth weight, 3,600 g), mild dehydration, mild hypotonia, and slight hyperpigmentation of the areolae and scrotum.

Brain magnetic resonance imaging, ambulatory electroencephalography, and cerebrospinal fluid examination showed no obvious abnormalities. Laboratory tests showed severe hyponatremia, with serum sodium as low as 88 mmol/L (reference range, 135–150 mmol/L), hypochloremia (57.6 mmol/L), hypomagnesemia, moderate metabolic acidosis (pH 7.439, BEb, −9.5 mmol/L; HCO_3_^−^, 11.2 mmol/L), and normal serum potassium, ammonia, and glucose levels. The renin–angiotensin–aldosterone profile showed renin of 9.26 ng/mL (reference range, 1.31–3.95), aldosterone of 577.05 pg/mL (reference range, 10–160), and angiotensin II of 340.16 pg/mL (reference range, 25–129). The 24-hour urinary calcium excretion was 0.33 mmol. The fractional excretion of magnesium was 10.9%. We administered 3% sodium chloride intravenously to increase the serum sodium level, with sodium supplementation of approximately 8–10 mmol/L per day. We also administered 25% magnesium sulfate (0.25 mL/kg/day) intramuscularly to increase serum magnesium levels. After 17-hydroxyprogesterone (17-OHP) and adrenocorticotrophic hormone (ACTH) results showed marked elevations (17-OHP, 740.2 ng/mL; reference range, <3; ACTH, 488.4 pg/mL; reference range, 7.2–63.3), we suspected 21-OHD. Glucocorticoid replacement therapy was initiated with hydrocortisone at 100 mg/m^2^/day. Serum potassium levels were mostly within the normal range, and hypomagnesemia was present on the second day of hospitalization. Therefore, after obtaining consent from the guardian, whole-exome sequencing and multiplex ligation-dependent probe amplification combined with long-range PCR were performed.

**Figure 1:**
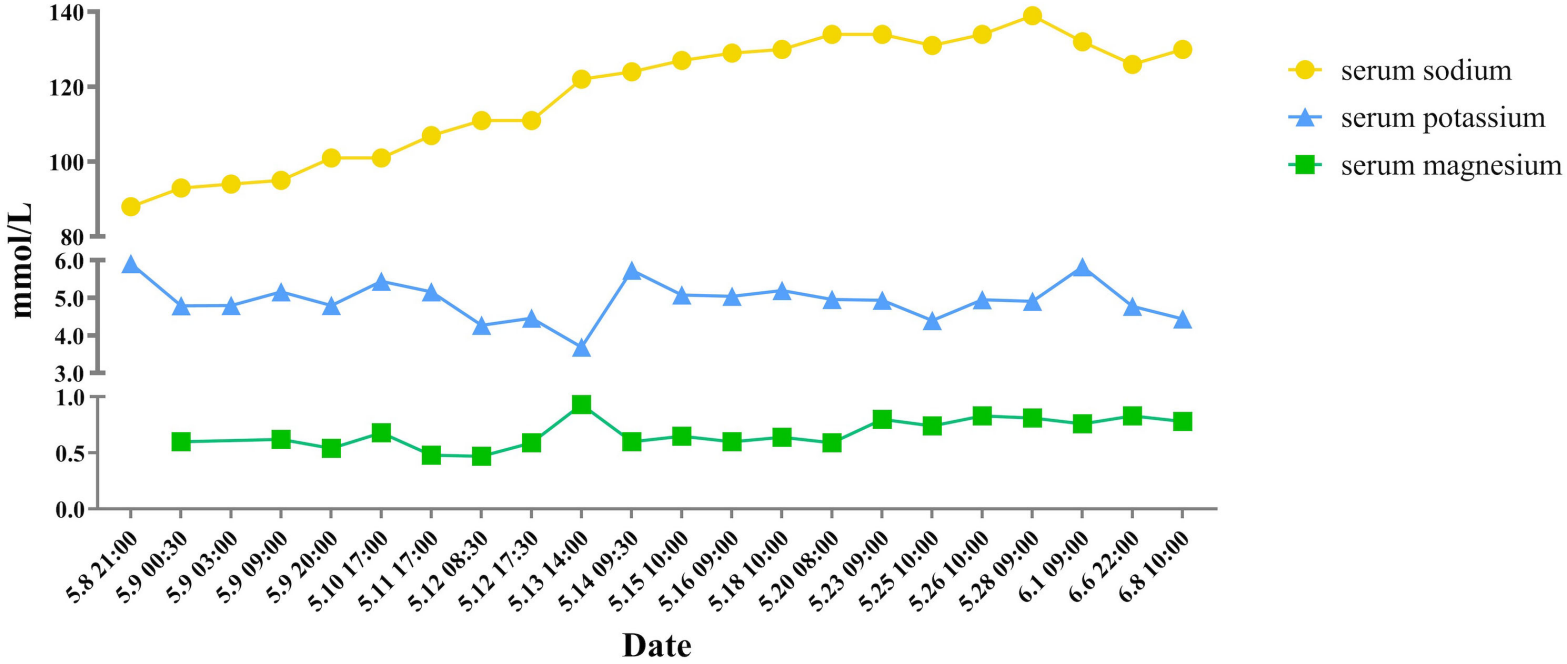
Trend chart of serum sodium, potassium, and magnesium levels. The x-axis represents the date and time of the electrolyte assay, and the y-axis represents the three electrolyte levels in mmol/L.

Gastrointestinal bleeding occurred on the second day of glucocorticoid treatment. We discontinued glucocorticoids, investigated the cause of gastrointestinal bleeding, and provided supportive treatment, including fasting, decompression, total parenteral nutrition, anti-infective therapy, hemostasis, acid suppression, and thrombin supplementation. After recovery from gastrointestinal bleeding, hydrocortisone was restarted at 20 mg/m^2^/day by intravenous infusion, subsequently changed to oral hydrocortisone (20 mg/m^2^/day) and then to fluhydrocortisone (0.05 mg/day). Serum sodium gradually returned to normal. Changes in electrolyte levels are shown in **Figure 1**. The patient was fed a lactose-free, protein hydrolyzed formula powder, and sodium chloride was added to the milk. The infant was then discharged from the hospital.

Genetic testing identified two pathogenic variants. One was a homozygous variant, 293-13C>G (p.)?, in intron 2 of the *CYP21A2* gene, resulting in an abnormal splicing variant and consistent with 21-OHD (OMIM: 201910). The other was a compound heterozygous variant, c.473G>A (p.Arg158Gln) and c.2720 + 3A>T (p.)?, in the *SLC12A3* gene, resulting in a missense and a splice variant, respectively, consistent with GS (OMIM: 263800).

21-OHD is caused by *CYP21A2* variants that result in 21-hydroxylase deficiency. 21-hydroxylase catalyzes the conversion of progesterone to deoxycorticosterone. Enzyme deficiency decreases deoxycorticosterone production, which reduces aldosterone. Aldosterone acts on the epithelial cells of the distal convoluted tubules and collecting ducts to increase potassium excretion and sodium reabsorption, leading to hyperkalemia and hyponatremia in aldosterone deficiency. GS is an autosomal recessive disorder caused by variants in *SLC12A3*, which encodes the thiazide diuretic-sensitive sodium–chloride cotransporter (NCC) in the distal convoluted tubule. *SLC12A3* variants impair NCC structure and function in the epithelial cells of the distal convoluted tubule, leading to abnormal sodium and chloride reabsorption, hypovolemia, renin–angiotensin–aldosterone system (RAAS) activation, hypokalemia, and metabolic alkalosis. Given that 21-OHD and GS have opposing effects on potassium metabolism, this infant did not develop marked hyperkalemia.

During outpatient follow-up, the child’s electrolyte levels remained normal, and his weight gradually increased. At 5 months of age, growth and development were normal, with good weight gain and normal electrolytes, and five sex hormone levels. 17-OHP level gradually declined, as shown in **Table 1**.

**Table 1:** Laboratory results and body weight during the follow-up period.

Time point after discharge	Na (mmol/L)	K (mmol/L)	Mg (mmol/L)	ACTH (pg/mL)	Cortisol (nmol/L)	17-OHP (ng/mL)	Five sex hormones	Weight (Kg)	Growth curve percentile
10 days	129	4.94	0.73					3.2	<P_3_
1 month	137	5.59	0.74	97.04	16	45	N	4.25	<P_3_
2 months	132	5.24	0.81					5.4	<P_3_
3 months	131	5.37	0.83					6.6	P_3_
4 months	138	5.46	0.89	74.14	135.2	7.8	N	7.5	P_10_

N indicates that the test result was within the reference range. Blank cells indicate that the test was not performed. Na indicates serum sodium. K indicates serum potassium. Mg indicates serum magnesium. 17-OHP indicates 17-hydroxyprogesterone. ACTH indicates adrenocorticotrophic hormone. P indicates the percentile of the growth curve.

## 3 Discussion

The patient presented with seizures in the neonatal period. After admission, laboratory tests identified severe hyponatremia as the cause. The serum sodium level was 88 mmol/L, and survival with such severe hyponatremia is rare. In the literature, the lowest reported serum sodium in an infant was 94 mmol/L (8), demonstrating that the serum sodium level in this case is extremely uncommon. The cause of hyponatremia should be assessed based on clinical manifestations, medical history, medication history, and laboratory findings. Hyponatremia is divided into normal blood volume hyponatremia, hypervolemic hyponatremia, and hypovolemic hyponatremia. Hypovolemic hyponatremia was considered given the child’s plasma osmolality of 200.3 mOsm/kg. Common causes of hypovolemic hyponatremia include prolonged diuretic use, osmotic diuresis, salt-wasting nephropathy, metabolic alkalosis, adrenal insufficiency, gastrointestinal loss, profuse sweating, and loss of the third space (9). Laboratory tests showed elevated testosterone, 17-OHP, and ACTH levels, and the child was clinically diagnosed with 21-OHD.

Notably, although the clinical diagnosis of 21-OHD was established, the child’s serum potassium was not significantly increased (maximum, 5.91 mmol/L), which was inconsistent with the severity of hyponatremia, and hypomagnesemia was present. These findings can be explained by the combined pathophysiology of the two disorders. 21-OHD is caused by *CYP21A2* variants that result in 21-hydroxylase deficiency. In this case, a homozygous variant, 293-13C>G (p.)?, in intron 2 of *CYP21A2* was detected by whole-exome sequencing, multiplex ligation-dependent probe amplification, and long-range PCR. As noted above, aldosterone deficiency can lead to hyponatremia and hyperkalemia. GS is an inherited tubulopathy resulting from variations in *SLC12A3*, which encodes the thiazide-sensitive NCC responsible for sodium chloride reabsorption in the early distal convoluted tubule (10). In this case, a compound heterozygous variant, c.473G>A (p.Arg158Gln) and c.2720 + 3A>T (p.)?, in *SLC12A3* was detected. These variants impair NCC structure and function, reducing sodium and chloride reabsorption, leading to hypovolemia, RAAS activation, hypokalemia, and metabolic alkalosis. Together, the opposing effects of 21-OHD and GS on electrolyte balance explain this infant’s severe hyponatremia, hypomagnesemia, and absence of marked hyperkalemia.

21-OHD is classified into three subtypes according to clinical severity: classic salt-wasting (SW), classic simple virilizing, and nonclassical (11). The clinical manifestations of SW include feeding difficulties, poor weight gain, electrolyte disturbances (hyponatremia and hyperkalemia), and metabolic acidosis (12), which are consistent with this patient. This case was classified as classic salt-wasting. GS is characterized by hypokalemic metabolic alkalosis, hypomagnesemia, and hypocalciuria with preserved kidney function and has substantial clinical heterogeneity (6). GS typically presents in adolescents and adults, partly in childhood, but is rarely reported in neonates (6, 7, 13–15). Sheng QQ et al. reported a cohort of 30 children with GS, and the minimum age of onset was 3 months in this study, which suggests that GS can occur in infancy (13). Pediatric GS presents with an insidious onset, and neonatal GS even have no obvious clinical manifestations. And the newborn cannot express their discomfort symptoms, when the blood potassium and magnesium are seriously decreased, the accumulation of myocardial, skeletal muscle and other conditions, will appear obvious symptoms and clinical manifestations, and then has reached the childhood or adolescent period. The literature reported a patient diagnosed with 21-OHD in the neonatal period had symptoms of hypokalemia at the age of 15 years (16). The clinician reevaluated the diagnosis and treatment of the patient, and the pathogenic gene of GS was found through gene detection. This literature showed that GS and 21-OHD could coexist in the same patient, except that there were no clinical manifestations of GS in the neonatal period. The infant in our report was diagnosed as GS mainly due to genetic diagnosis. The infant presented with hypomagnesemia and increased fractional excretion of magnesium, which was consistent with the clinical manifestations of GS.

Therefore, when clinical phenotypic heterogeneity is suspected and the diagnosis does not fully explain the presentation, genetic testing should be considered to confirm the diagnosis and support early intervention, early treatment, and individualized management. Both CAH and GS are chronic diseases; long-term follow-up, multidisciplinary care, and individualized treatment are crucial. The child was followed up to 5 months of age and had normal growth and development, good weight gain, and normal electrolytes, five sex hormone levels, and 17-OHP.

## 4 Conclusions

Severe hyponatremia is extremely rare in newborns. In this case, the infant was diagnosed early with coexisting 21-OHD and GS, two conditions that have opposing effects on potassium balance (hyperkalemia in 21-OHD and hypokalemia in GS). Accordingly, the infant’s atypical phenotype, including near-normal serum potassium, moderate metabolic acidosis, and hypomagnesemia, can thus be explained. This case report provides diagnostic and treatment insights for clinicians and highlights the value of genetic testing when the diagnosis is uncertain.

## Data Availability

The original contributions presented in the study are included in the article/supplementary material. Further inquiries can be directed to the corresponding author/s.
